# Sterile Protection against Malaria Is Independent of Immune Responses to the Circumsporozoite Protein

**DOI:** 10.1371/journal.pone.0001371

**Published:** 2007-12-26

**Authors:** Anne Charlotte Grüner, Marjorie Mauduit, Rita Tewari, Jackeline F. Romero, Nadya Depinay, Michèle Kayibanda, Eliette Lallemand, Jean-Marc Chavatte, Andrea Crisanti, Photini Sinnis, Dominique Mazier, Giampietro Corradin, Georges Snounou, Laurent Rénia

**Affiliations:** 1 Institut Cochin, Department of Immunology, Université Paris Descartes, Centre National de la Recherche Scientifique (CNRS) UMR 8104, Paris, France; 2 Inserm, U567, Paris, France; 3 Division of Cell and Molecular Biology, Faculty of Natural Sciences, Imperial College London, London, United Kingdom; 4 Institut de Biochimie, Université de Lausanne, Epalinges, Lausanne, Switzerland; 5 Equipe Parasitologie Comparée et Modèles Expérimentaux USM0307, Centre National de la Recherche Scientifique (CNRS) IFR101, Muséum National d'Histoire Naturelle, Paris, France; 6 Department of Medical Parasitology, New York University School of Medicine, New York, New York, United States of America; 7 Université Pierre et Marie Curie-Paris 6, UMR S511 Paris, France; 8 INSERM, U511, Paris, France; 9 Assistance Publique - Hôpitaux de Paris (AP-HP), Groupe hospitalier Pitié-Salpêtrière, Service Parasitologie-Mycologie, Paris, France; 10 Singapore Immunology Network, Agency for Science, Technology and Research (A*STAR), Biopolis, Singapore; Federal University of Säo Paulo, Brazil

## Abstract

**Background:**

Research aimed at developing vaccines against infectious diseases generally seeks to induce robust immune responses to immunodominant antigens. This approach has led to a number of efficient bacterial and viral vaccines, but it has yet to do so for parasitic pathogens. For malaria, a disease of global importance due to infection by *Plasmodium* protozoa, immunization with radiation-attenuated sporozoites uniquely leads to long lasting sterile immunity against infection. The circumsporozoite protein (CSP), an important component of the sporozoite's surface, remains the leading candidate antigen for vaccines targeting the parasite's pre-erythrocytic stages. Difficulties in developing CSP-based vaccines that reproduce the levels of protection afforded by radiation-attenuated sporozoites have led us to question the role of CSP in the acquisition of sterile immunity. We have used a parasite transgenic for the CSP because it allowed us to test whether a major immunodominant *Plasmodium* antigen is indeed needed for the induction of sterile protective immunity against infection.

**Methodology/Main Findings:**

We employed a *P. berghei* parasite line that expresses a heterologous CSP from *P. falciparum* in order to assess the role of the CSP in the protection conferred by vaccination with radiation-attenuated *P. berghei* parasites. Our data demonstrated that sterile immunity could be obtained despite the absence of immune responses specific to the CSP expressed by the parasite used for challenge.

**Conclusions:**

We conclude that other pre-erythrocytic parasite antigens, possibly hitherto uncharacterised, can be targeted to induce sterile immunity against malaria. From a broader perspective, our results raise the question as to whether immunodominant parasite antigens should be the favoured targets for vaccine development.

## Introduction

An ideal target of vaccination against malaria would be the initial PE stages of the infection, i.e. the sporozoite and the hepatic phase. Sporozoites inoculated by the mosquito can only invade and develop within hepatocytes to generate merozoites that, once released in the blood stream, initiate the pathogenic erythrocytic phase. Effective inhibition of this obligatory and transient phase of the life cycle would prevent infection, disease, and reduce transmission. Sterile immunity against PE stages is an all-or-none phenomenon, since successful infection of a single hepatocyte would lead to a patent blood infection. In humans, immunization with large numbers of radiation-attenuated sporozoites remains the only protocol that leads to the induction of sterile immunity [1], [2]. Subsequent investigations revealed a role for both humoral and cellular immune responses targeting the sporozoite and the infected hepatocyte, respectively^3^. Until recently, only a few antigens thought to be implicated in protection against the PE stages had been identified (CSP, Liver Stage Antigens 1 & 3, and the Thrombospondin Related Anonymous Protein)[3].

The dominant antibody responses directed against the antigenic repetitive central domain of CSP in the vaccinated hosts [4], and the ability of passively transferred CSP-specific CD8^+^ and CD4^+^ T cell clones to fully protect against sporozoite challenge [5]–[7], have led to consider the CSP as the most likely parasite antigen implicated in the sterile protection induced by irradiated sporozoites. Since the mid-1980s numerous experimental subunit vaccines based solely or partially on CSP have been tried in naïve volunteers and endemic residents [8]. However, when vaccination trials using the latest and most advanced CSP-based formulation, RTS,S, were conducted in endemic residents, the extent of the sterile protection observed was moderate and transient in nature [9]–[11]. The modest efficacies of this and other experimental CSP-based vaccines, led us to question the importance of immune responses against CSP in the induction and acquisition of sterile protection. There are immunological and epidemiological data that are consistent with a peripheral role for CSP in naturally acquired protective responses. First, CSP-specific immune responses in naturally exposed humans were found to be poorly predictive of protection [12]. Second, molecular epidemiological observations suggested that immune responses to CSP do not exert a strong selective pressure [13], [14]. Third, analyses of immune responses in a subset of volunteers protected by immunization with irradiated sporozoites revealed that the predominant cellular responses were directed at parasite antigens other than CSP [15]. For ethical reasons, proof for the involvement of CSP in sterile protection can only be derived from animal models. Formal demonstration of the role of CSP in the induction or sterile immunity cannot be obtained by knock-out studies since the protein is necessary for the development of infective sporozoites [16]. Recent advances in transgenic technology afforded a means to assess the actual contribution of CSP in the acquisition of sterile immunity following immunization of mice with irradiated *P. berghei* sporozoites. To this end, we used a recently described transgenic *P. berghei* line, *P. berghei* [*PfCS*], whose CSP gene was replaced by that of *P. falciparum* [17]. The *P. berghei* [*PfCS*] parasites had been previously shown to produce functional infective sporozoites [17].

## Materials and Methods

### Mice and *Plasmodium* parasites

BALB/cJ or [BALB/c×C57BL/6J] F1 mice were purchased from Harlan Laboratories (Gannat, France), and were housed in a pathogen-free rodent barrier facility. All experiments and procedures were performed in compliance with institutional and national guidelines. *P. berghei* ANKA cloned lines transfected either with the *P. falciparum* CSP gene from the Welcome strain [17] or with a GFP gene [18] both of which had been submitted to the same selection procedure, were used to infect laboratory-bred *Anopheles stephensi* mosquitoes. Sporozoites from the two parasite lines were obtained by dissection of infected *A. stephensi* salivary glands 17 to 21 days after the infective blood meal.

### Immunization and challenge

In order to induce sterile immunity in all the animals, BALB/cJ mice were immunized with 12 000 rad-irradiated *P. berghei* sporozoites as follows: one dose of 75 000 sporozoites followed by two booster doses of 25 00 of *P. berghei* sporozoites on days 15 and 21. In [BALB/c×C57BL/6] F1 mice immunisation was made with 3 injections of 10 000 *P. berghei* irradiated sporozoites at days 0, 15 and 21. Control and mice irradiated sporozoites-immunized mice were challenged intravenously with 5 000 *P. berghei* or *P. berghei* [*PfCS*] sporozoites. Blood stage infection was determined by the presence of parasites in Giemsa-stained blood smears prepared daily from days 3 to 10 post-challenge, and parasitaemia was determined by counting the number of infected red blood cells per 1000 erythrocytes.

### Peptides

The following peptides were synthesized by one of us (GC): a) peptides that correspond to the repeat region of the *P. berghei* or the *P. falciparum* CS protein, (DPPPPNPN)_2_ and NANP_50_, respectively, were used in ELISA as previously described [19]–[22]. Lyophilized material was resuspended in sterile distilled water at 10 mg/ml, aliquoted, and stored at - 20°C until use, b) long peptides that spanned the NH_2_-terminal and COOH-terminal of the *P. berghei* CSP, PbNt (GYGQNKSIQA QRNLNELCYN EGNDNKLYHV LNSKNGKIYI RNTVNRLLAD APEGKKNEKK NEKIERNNKL K) and PbCt (NDDSYIPSAE KILEFVKQIR DSITEEWSQC NVTCGSGIRV RKRKGSNKKA EDLTLEDIDT EICKMDKCS), respectively, and c) long peptides that spanned the NH_2_-terminal and COOH-terminal of the *P. falciparum* CSP, PfNt (YQCYGSSSNT RVLNELNYDN AGTNLYNELE MNYYGKQENW YSLKKNSRSL GENDDGNNNN GDNGREGKDE DKRDGNNEDN EKLRKPKHKK LKQPGDGNPD PNA) and PfCt (KNNQGNGQGH NMPNDPNRNV DENANANNAV KNNNNEEPSD KHIEQYLKKI KNSISTEWSP CSVTCGNGIQ VRIKPGSANK PKDELDYEND IEKKICKMEK CS) [20].

### ELISA

The presence and level of antibodies to peptides corresponding to the repeat region of the *P. berghei*, (DPPPPNPN)_2_, and *P. falciparum*, NANP_50_, CSP proteins, or to the long peptides that span the N- and C-termini of the CSP proteins from these two parasite species, were determined by ELISA as described previously [23]. Briefly, 96-well flat-bottom plates (Maxisorp, Nunc, Roskilde, Denmark) were coated with 1 µg/ml in PBS, pH 7.8, by overnight incubation at 4°C. After extensive washes, the wells were blocked with 200 µl of PBS-Tween (PBS containing 0.05% Tween and 1% bovine serum albumin) for 1 h. The wells were incubated overnight at 4°C with 100 µl mouse sera diluted 1/100 in PBS-Tween, then washed twice and incubated for 45 min at room temperature, either with a Goat anti-mouse IgM (Invitrogen SARL, Cergy Pontoise, France) or with a biotinylated-goat anti-mouse IgG (Jackson ImunoResearch Europe Ltd, Newmarket, United Kingdom) diluted in PBS-Tween. The wells containing the anti-IgM antibody were washed and further incubated with a biotinylated rabbit anti-goat IgG (Sigma-Aldrich, Saint-Quentin Fallavier, France) diluted in PBS-Tween for 45 min at room temperature, then washed and incubated with extravidin-coupled alkaline phosphatase (Sigma-Aldrich) diluted in PBS-Tween 1h at room temperature. Phosphatase activity was measured using 4-methylumbelliferyl phosphate (Sigma-Aldrich) as a substrate and the fluorescence at 355/460 nm was measured using a spectrophotometer (Victor 1420, Wallac Oy, Turku, Finland).

### Immunofluorescence assay

Sera from mice immunized with irradiated sporozoites were tested by immunofluorescence using air-dried methanol-fixed or unfixed (“wet”) sporozoites from the different *Plasmodium* lines, in order to detect total or surface antigen content as previously described [24].

### ELISPOT assay

PVDF microplates (Millipore, Bedford, MA, USA) were coated overnight at 4°C with 15 µg/ml of an anti-mouse IFN-γ rat monoclonal antibody (clone AN18, Mabtech AB, Sophia Antipolis, France) diluted in PBS. After extensive washes, the wells were blocked with RPMI medium containing 10% foetal calf serum for 2 h at 37°C. Spleen cells were incubated overnight with one of the peptides corresponding to a specific epitope (final concentration 10 µg/ml) and with 30 U/ml of recombinant human IL2. The plates were then washed, incubated with 2 µg/ml of biotinylated anti-mouse IFN-γ rat monoclonal antibody (clone R4-6A2, Mabtech AB) diluted in PBS containing 0.5% bovine serum albumin for 2 h at 37°C, and then overnight at 4°C. Plates were subsequently incubated with extravidin-coupled alkaline phosphatase (Sigma-Aldrich) diluted in PBS. After adding the BCIP/NBT substrate (Sigma-Aldrich), IFN-γ spot forming cells (SFC) were counted under a stereomicroscope and expressed as the number of spots per million cells tested.

## Results and Discussion

**Figure 1:**
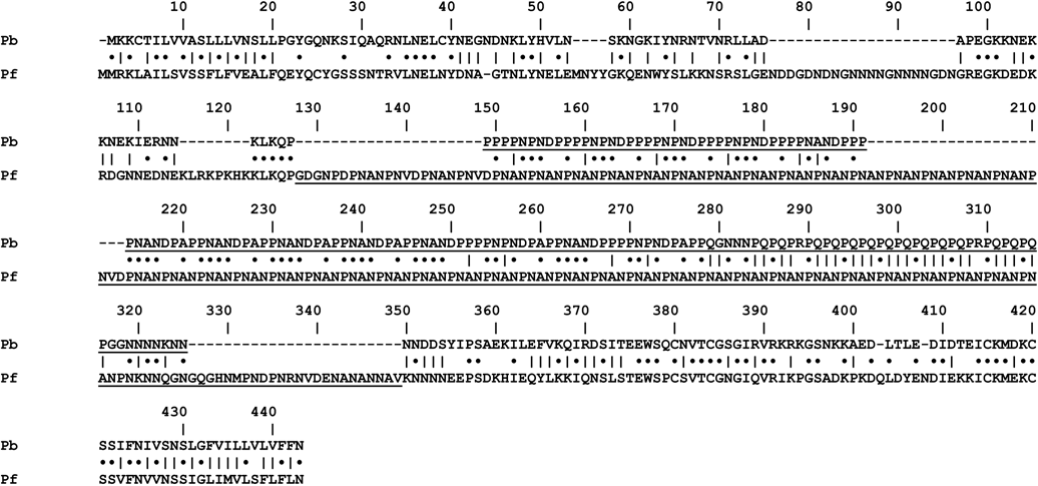
Comparison of the amino acid sequences of the *P. berghei* ANKA clone cy17 (34) and *P. falciparum* Welcome strain (35) CSP polypeptides. Black dots represent identical amino acid residues while bars represent amino acid residues with similar characteristics. The repeat regions (including the pre- and post-repeat sequences) are underlined.

Given the major differences between the sequences of the *P. berghei* and *P. falciparum* CSP genes (Figure 1), we predicted that the immune responses induced by immunisation with irradiated sporozoites of the wild type *P. berghei* will be specific to the *P. berghei* CSP. In this case, any protection observed in the immunised mice challenged with the *P. berghei* [*PfCS*] parasites, would be due to immune responses independent of CSP.

**Figure 2:**
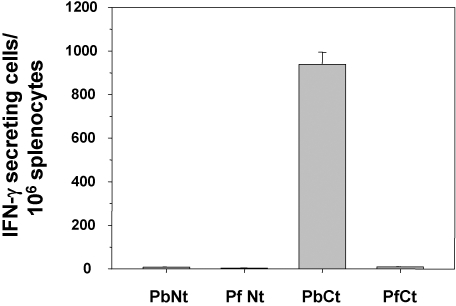
CSP-specific T cells induced by immunization with irradiated *P. berghei* sporozoites do not cross-react with the *P. falciparum* CSP expressed by the *P. berghei* [*PfCS*]. IFN-γ ELISPOT was used to determine the frequency of epitope-specific T cells in the spleens of immunised animals, using long peptides corresponding to the N-terminus (PbNt or PfNt) or the C-terminus (PbCt or PfCt) of *P. berghei* and *P. falciparum*, respectively. Results are expressed as the mean ± SEM of T cells from groups of 5 mice.

**Figure 3:**
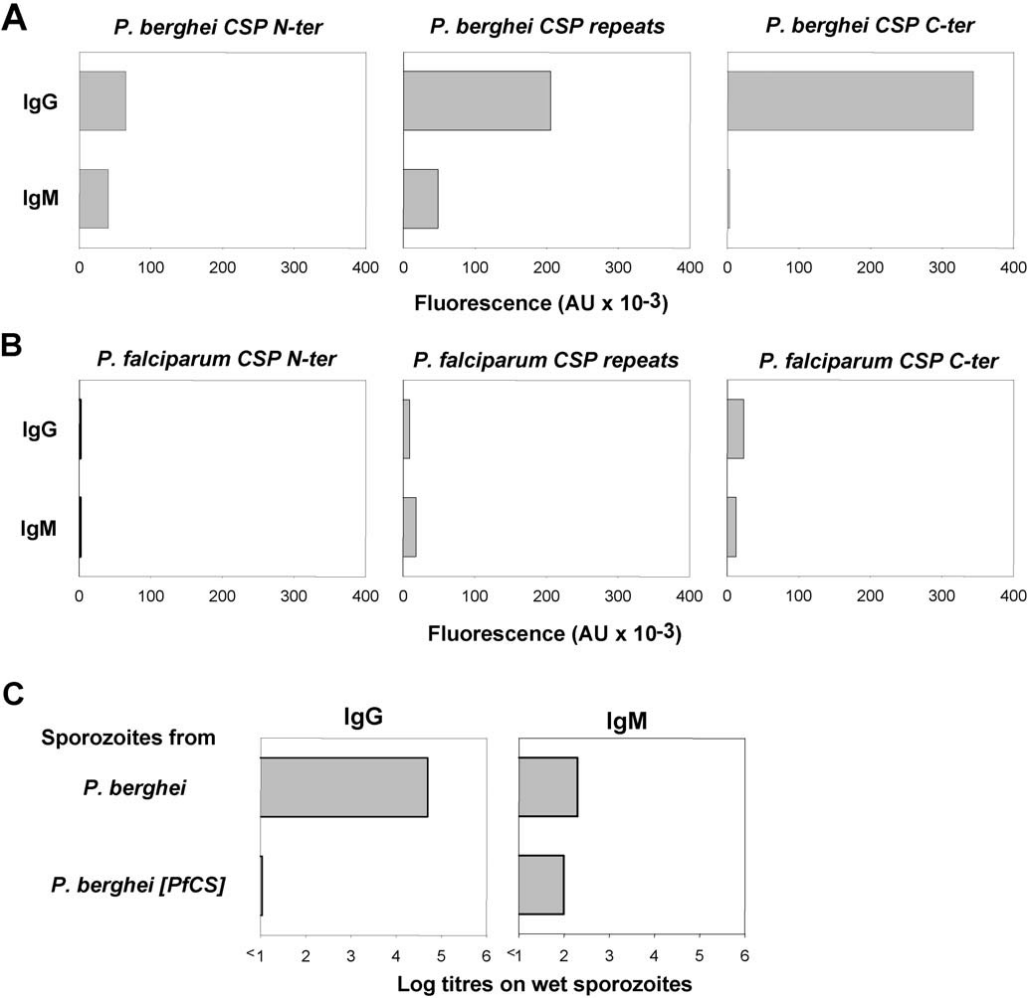
Antibody reactivity induced by immunization with irradiated sporozoites. Pooled serum samples from groups of mice immunized with sporozoites from the different parasite lines were analyzed by ELISA to assay IgG and IgM responses induced against *P. berghei* CSP (A), or *P. falciparum* CSP (B), using long peptides covering the N-terminus or the C-terminus of the antigen, and short peptides that include some of the repeat units of the central repetitive region. Serums were also tested by IFAT against wet sporozoites to detect anti-CSP IgG and IgM antibodies(C). Titres are expressed as the log of the highest dilution of serum giving a positive staining.

Groups of mice were immunized with three doses of irradiated *P. berghei* sporozoites. Sterile protection in this gold standard model is considered to depend on a combination of the antibody responses induced against CSP, leading to sporozoite inactivation and inhibition of hepatocyte invasion, and on the cellular responses induced against CSP, leading to the destruction of infected hepatocytes. Therefore, the immune responses induced against the CSP in the immunised mice were carefully characterized. A set of long peptides spanning all the CD4 and CD8 epitopes present in the N- and C-terminal regions flanking the central repeats of the *P. berghei* CSP [20], [21] or the *P. falciparum* CSP [19], [20], [22] were used to demonstrate that the T cell responses induced by immunization with *P. berghei* were minimally cross-reactive with the *P. falciparum* CSP (Figure 2). The IgG and IgM antibodies induced by immunisation with *P. berghei* irradiated sporozoites against the immunodominant central repeat region of CSP were then assayed. Cross-reactive anti-CSP antibodies to the *P. falciparum* CSP were undetectable or very low when assayed by ELISA using peptides specific to the repeat regions of the two proteins (Figures 2A and 2B). The bulk of the IgG response directed against the homologous *P. berghei* sporozoites was restricted to the CSP, corresponding to an IFAT titre of ∼1/51200 on wet sporozoites, and showed no reactivity of the heterologous *P. berghei* [*PfCS*] sporozoites with titres equal or below 1/10 (Figure 2C). The levels of the IgM responses induced were much lower than those of the IgG responses (Figure 2A), and most of the cross-reactive IgM responses, IFAT titres of 1/100, were directed against components other than the CSP (Figures 3B and 3C). The antibody responses in animals immunised only once with *P. berghei* irradiated sporozoites were much lower, nonetheless the same pattern of antibody reactivities was observed when their sera were similarly assayed (data not shown).

**Figure 4:**
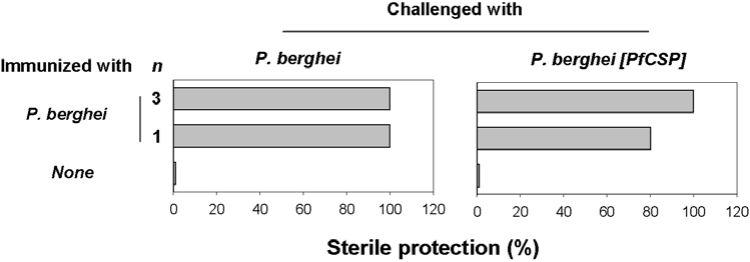
Sterile protection in mice immunized with *P. berghei* irradiated sporozoites and challenged with *P. berghei* or *P. berghei* [*PfCS*] sporozoites. Mice were immunized with 1 or 3 injections of *P. berghei* (indicated on the left of the panel) before challenge with 5 000 *P. berghei* or *P. berghei* [*PfCS*] sporozoites. All naive control mice developed a patent blood-stage infection. The data are representative of those obtained in duplicate experiments.

**Figure 5:**
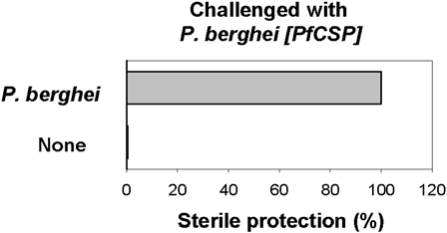
Sterile protection in [BALB/c×C57BL/6] F1 mice immunized three times with *P. berghei* or *P. berghei* [*PfCS*] and challenged with 5000 *P. berghei* or *P. berghei* [*PfCS*] sporozoites. All animal groups (5 mice per group) were monitored for blood-stage infections by examination of Giemsa-stained blood smears obtained daily from day 2 to day 11 post-challenge. All naive control mice developed a patent blood-stage infection.

Since minimal CSP cross-reactive responses are induced by immunisation with wild type *P. berghei* irradiated sporozoites, if the sterile protection induced were dependent on CSP-specific immune responses, then the immunized mice should not be protected against challenge with *P. berghei* [*PfCS*] sporozoites that express a heterologous CSP. However, BALB/c mice immunized, either once or three times, with *P. berghei* irradiated sporozoites were found to be protected from infection whether challenged with homologous sporozoites or with those from *P. berghei* [*PfCS*] (Figure 4). These observations were not restricted to BALB/c mice since [BALB/c×C57BL/6] F1 mice immunized three times with *P. berghei* irradiated sporozoites and challenged with *P. berghei* [*PfCS*] were also protected (Figure 5). These results demonstrate that the acquisition of sterile immunity can be achieved independently of the CSP-specific cellular and humoral responses induced by immunization. Thus, it can be concluded that the contribution of CSP is not essential to sterile protection.

Recently, a series of elegant experiments based on mice made tolerant to the CSP of *P. yoelii*, another rodent malaria model [25] led to deduce an immunodominant protective role for CSP, an interpretation that contradicts the conclusions reported here. In these experiments, the weight of CSP in protection was defined in terms of reduced hepatic parasite burden (as measured by quantitative RT-PCR), a measure not necessarily indicative of sterile protection. Parasite burdens in CSP-tolerant mice immunized with two doses of irradiated sporozoites were indeed 10 000 times higher than those observed in similarly treated control animals. Nonetheless, when 3 immunizing doses were used, as was the case in the investigations reported here, full protection against sporozoite challenge was observed in the mice made tolerant to the CSP, thus casting a doubt on a central role for CSP in sterile immunity. Two possible reasons were put forward to account for this key observation [26]: first, a breakdown in tolerance to CSP by the third immunizing dose, or second, a preponderant role for non-CSP parasite antigens. The second view is consistent with the powerful CD8^+^ protective responses directed against non-CSP antigens demonstrated in the CSP-tolerant mice [25], as well as with our experimental observations obtained using normal mice where the issue of immune tolerance does not apply.

In conclusion, despite the B cell immunodominance of CSP, we demonstrate that the CSP-specific responses induced by immunization with irradiated sporozoites are not necessary to account for the gold standard anti-malaria protection, i.e. prevention of the blood stage infection. The fact that CSP is the major surface protein of sporozoites which it covers entirely, associated to the highly biased antibody responses directed against the sporozoite, suggest that the CSP might play a significant role in immune evasion: by monopolizing the host responses mounted against PE parasites, the CSP would deviate the host defences away from other antigens capable of acting as targets of the sterile protective immunity, such as the one induced by immunization with irradiated sporozoites. It is acknowledged that the conclusions derived from investigations into the immunity against PE stages in the rodent models are applicable to the situation in the human infection [27], [28]. Thus our data suggest that immunity induced against *P. falciparum* CSP in humans might also be inadequate in conferring sterile protection. This would explain the limited efficacy of sub-unit CSP based vaccines [11]. Given the major costs associated with the development and clinical testing of vaccines against the pre-erythrocytic stages of *P. falciparum*, it might be judicious to adopt the strategy presented here to other candidates for which a homologue exists in parasites species that infect rodents. Moreover, the search for the parasite antigens that are actually implicated in the acquisition of sterile protection should be strongly encouraged. Finally, our data illustrates the value of using whole parasites in studies aimed at elucidating malaria immunity, to the development of synthetic, subunit, or practical live sporozoite vaccination strategies [29]–[33].

## References

[1] Nussenzweig RS, Vanderberg JP, Most H, Orton CG (1967). Protective immunity produced by injection of X-irradiated sporozoites of *Plasmodium berghei.*. Nature.

[2] Hoffman SL, Goh LM, Luke TC, Schneider I, Le TP (2002). Protection of humans against malaria by immunization with radiation-attenuated *Plasmodium falciparum* sporozoites.. J Infect Dis.

[3] Druilhe P, Rénia L, Fidock DA, Sherman IW (1998). Malaria: Parasite Biology, Pathogenesis, and Protection,.

[4] Romero P, Tam JP, Schlesinger DH, Clavijo P, Gibson H (1988). Multiple T helper cell epitopes of the circumsporozoite protein of *Plasmodium berghei.*. Eur J Immunol.

[5] Romero P, Maryanski JL, Corradin G, Nussenzweig RS, Nussenzweig V, Zavala F (1989). Cloned cytotoxic T cells recognize an epitope on the circumsporozoite protein and protect against malaria.. Nature.

[6] Rénia L, Grillot DA, Marussig M, Corradin G, Miltgen F (1993). Effector functions of circumsporozoite peptide-primed CD4^+^ T cell clones against *Plasmodium yoelii* liver stages.. J Immunol.

[7] Rodrigues MM, Cordey AS, Arreaza G, Corradin G, Romero P (1991). CD8^+^ cytolytic T cell clones derived against the *Plasmodium yoelii* circumsporozoite protein protect against malaria.. Int Immunol.

[8] Hill AVS (2006). Pre-erythrocytic malaria vaccines: towards greater efficacy.. Nat Rev Immunol.

[9] Bojang KA, Milligan PJM, Pinder M, Vigneron L, Alloueche A (2001). Efficacy of RTS,S/AS02 malaria vaccine against *Plasmodium falciparum* infection in semi-immune adult men in The Gambia: a randomised trial.. Lancet.

[10] Alonso PL, Sacarlal J, Aponte JJ, Leach A, Macete E (2004). Efficacy of the RTS,S/AS02A vaccine against *Plasmodium falciparum* infection and disease in young African children: randomised controlled trial.. Lancet.

[11] Snounou G, Gruner AC, Muller-Graf CD, Mazier D, Renia L (2005). The *Plasmodium* sporozoite survives RTS,S vaccination.. Trends in Parasitology.

[12] Hoffman SL, Oster CN, Plowe CV, Woollet GR, Beier JC (1987). Naturally acquired antibodies to sporozoites do not prevent malaria: vaccine development implications.. Science.

[13] Kumkhaek G, Phra-ek K, Renia L, Singhasivanon P, Looareesuwan S (2005). J Immunol.

[14] Weedall GD, Preston BMJ, Thomas AW, Sutherland CJ, Conway DJ (2007). Differential evidence of natural selection on two leading sporozoite stage malaria vaccine candidate antigens.. Int J Parasitol.

[15] Doolan DL, Southwood S, Freilich DA, Sidney J, Graber NL (2003). Identification of *Plasmodium falciparum* antigens by antigenic analysis of genomic and proteomic data.. Proc Natl Acad Sci USA.

[16] Ménard R, Sultan AA, Cortes C, Altszuler R, van Dijk MR (1997). Circumsporozoite protein is required for development of malaria sporozoites in mosquitoes.. Nature.

[17] Tewari R, Scacapelo R, Bistoni F, Holder AA, Crisanti A (2002). Function of region I and II adhesive motifs of *Plasmodium* circumsporozoite protein in sporozoite motility and infectivity.. J Biol Chem.

[18] Franke-Fayard B, Trueman H, Ramesar J, Mendoza J, van der Keur M (2004). A *Plasmodium berghei* reference line that constitutively expresses GFP at a high level throughout the complete life cycle.. Mol Biochem Parasitol.

[19] Roggero MA, Filippi B, Church P, Hoffman SL, Blum-Tirouvanziam U (1995). Synthesis and immunological characterization of 104-mer and 102- mer peptides corresponding to the N- and C-terminal regions of the *Plasmodium falciparum* CS protein.. Mol Immunol.

[20] Eberl G, Renggli J, Men Y, Roggero MA, Lopez JA, Corradin G (1999). Extracellular processing and presentation of a 69-mer synthetic polypeptide to MHC class I-restricted T cells.. Mol Immunol.

[21] Roggero MA, Meraldi V, Lopez JA, Eberl G, Romero JC (2000). The synthetic, oxidized C-terminal fragment of the *Plasmodium berghei* circumsporozoite protein elicits a high protective response.. Eur J Immunol.

[22] Lopez JA, Weilenman C, Audran R, Roggero MA, Bonelo A (2001). A synthetic malaria vaccine elicits a potent CD8^+^ and CD4^+^ T lymphocyte immune response in humans. Implications for vaccination strategies.. Eur J Immunol.

[23] Grillot D, Michel M, Muller I, Tougne C, Renia L (1990). Immune responses to defined epitopes of the circumsporozoite protein of the murine malaria parasite, *Plasmodium yoelii.*. Eur J Immunol.

[24] Rénia L, Miltgen F, Charoenvit Y, Ponnudurai T, Verhave JP (1988). Malaria sporozoite penetration: a new approach by double staining.. J Immunol Meth.

[25] Kumar KA, Sano G, Boscardin S, Nussenzweig RS, Nussenzweig MC (2006). The circumsporozoite protein is an immunodominant protective antigen in irradiated sporozoites.. Nature.

[26] Hoffman SL (2006). Malaria: a protective paradox.. Nature.

[27] Hoffman SL, Rogers WO, Carucci DJ, Venter JC (1998). From genomics to vaccines: malaria as a model system.. Nature Med.

[28] Nussenzweig RS, Zavala F (1997). A malaria vaccine based on a sporozoite antigen.. N Engl J Med.

[29] Mueller AK, Labaied M, Kappe SH, Matuschewski K (2005). Genetically modified *Plasmodium* parasites as a protective experimental malaria vaccine.. Nature.

[30] Mueller AK, Camargo N, Kaiser K, Andorfer C, Frevert U (2005). *Plasmodium* liver stage developmental arrest by depletion of a protein at the parasite-host interface.. Proceedings of the National Academy of Sciences USA.

[31] van Dijk MR, Douradinha B, Franke-Fayard B, Heussler V, van Dooren MW (2005). Genetically attenuated, P36p-deficient malarial sporozoites induce protective immunity and apoptosis of infected liver cells.. Proceedings of the National Academy of Sciences USA.

[32] Luke TC, Hoffman SL (2003). Rationale and plans for developing a non-replicating, metabolically active, radiation-attenuated *Plasmodium falciparum* sporozoite vaccine.. J Exp Biol.

[33] Rénia L, Grüner AC, Mauduit M, Snounou G (2006). Vaccination against malaria with live parasites.. Expert Rev Vaccines.

[34] Hall N, Karras M, Raine JD, Carlton JMR, Kooij TW (2005). A comprehensive survey of the *Plasmodium* life cycle by genomic, transcriptomic, and proteomic analyses.. Science.

[35] Lockyer MJ, Schwartz RT (1987). Strain variation in the circumsporozoite protein of *Plasmodium falciparum*.. Mol Biochem Parasitol.

